# Exploring the mediating role of the non-high-density lipoprotein cholesterol to high-density lipoprotein cholesterol ratio (NHHR) in the association between obesity and sleep-disordered breathing

**DOI:** 10.1007/s40519-025-01717-4

**Published:** 2025-02-02

**Authors:** Ying Cui, Ziyi Cheng

**Affiliations:** 1 Department of Public Health Science, Graduate School and Transdisciplinary Major in Learning Health Systems, Graduate School, Korea University, 145, Anam-Ro, Seongbuk-Gu, Seoul, Republic of Korea; 2 Department of Electrical and Electronic Engineering, The University of Manchester, Manchester, M13 9PL UK

**Keywords:** Sleep-disordered breathing, High-density lipoprotein cholesterol ratio, Body mass index, Restricted cubic spline model, Boruta algorithm

## Abstract

**Purpose:**

Exploring novel mediators affecting the relationship between obesity and sleep-disordered breathing (SDB) is necessary. This study aimed to explore the mediating role of the non-high-density lipoprotein cholesterol to high-density lipoprotein cholesterol ratio (NHHR) in the association between body mass index (BMI) and SDB using data from the 2015–2018 National Health and Nutrition Examination Survey (NHANES) cycles.

**Methods:**

Total 7639 participants were included. SDB was defined based on the self-reported frequency of snoring, snorting, or excessive daytime sleepiness. The BMI and NHHR were calculated based on height and weight measurements and laboratory data, respectively. Weighted multivariate logistic and linear regression analyses were conducted to examine the associations, and restricted cubic spline (RCS) analysis was used to assess dose–response relationships. Mediation analysis was performed to evaluate the NHHR’s role in the BMI–SDB association. Subgroup analyses were performed to assess differences across various populations.

**Results:**

SDB symptoms were observed in 51.05% of participants. Higher BMI was significantly associated with increased SDB risk. RCS analysis revealed a nonlinear relationship between BMI and SDB. Subgroup analyses indicated a positive correlation between BMI and SDB was stronger among nonhypertensive participants. NHHR was positively associated with BMI and SDB. Mediation analysis showed that the NHHR explained 5.44–8.12% of the BMI–SDB association.

**Conclusions:**

BMI is a critical factor in the risk of SDB, and the NHHR partially mediates this relationship. BMI and cholesterol levels should be managed to mitigate the SDB risk.

**Level of evidence:**

Level V—cross-sectional observational study.

## Background

Sleep-disordered breathing (SDB), a significant research area in sleep medicine, is characterised by irregular breathing patterns and insufficient ventilation during sleep, leading to intermittent hypoxia and sleep fragmentation [1, 2]. SDB is associated with various adverse health conditions, including cardiovascular diseases [3, 4], metabolic disorders, such as impaired glucose metabolism and insulin resistance [5, 6], and mortality [7]. In the United States, 18 million adults have SDB, which remains undiagnosed in many of them [8]. Clinically, the overall prevalence of significant SDB is as high as 32.7% [9], making SDB a major public health concern.

Obesity, typically evaluated based on body mass index (BMI), is a recognised risk factor for SDB [10]. Excess weight, typically defined as a body mass index (BMI) above 25 kg/m^2^ or an abnormal accumulation of adipose tissue, especially around the upper airway, can lead to airway narrowing, increasing the likelihood of airway collapse during sleep [11, 12]. Although the close relationship between high BMI and SDB severity is well established, the underlying mechanisms and mediating factors remain unclear. There is an urgent need to explore novel mediators affecting the relationship between obesity and SDB to pave the way for more effective intervention strategies.

Abnormal cholesterol levels, particularly dyslipidaemia, also play a significant role in the pathogenesis of SDB. Elevated cholesterol levels can lead to atherosclerosis and exacerbate SDB symptoms [13, 14]. In addition, high cholesterol levels may affect vascular elasticity and function, resulting in poor blood flow, further affecting normal respiratory system functioning [15, 16]. The non-high-density lipoprotein cholesterol to high-density lipoprotein cholesterol (HDL-C) ratio (NHHR) reflects the balance between atherogenic and protective lipoproteins and is an important indicator of the risk of diabetes and metabolic health [17]. However, evidence regarding the potential role of the NHHR in the relationship between obesity and SDB remains limited. As a novel approach, in this study, we considered the NHHR to be a key mediating factor in this relationship. Furthermore, we considered that an investigation of the NHHR would provide new insights into how obesity-induced changes in lipid profiles lead to SDB onset and progression. This novel approach would not only deepen our understanding of SDB but would also open new avenues for targeted therapeutic interventions.

Therefore, this study aimed to explore the mediating role of the NHHR in the relationship between BMI and SDB using the National Health and Nutrition Examination Survey (NHANES) database. The objective was to provide new evidence on the interactions among obesity, NHHR, and SDB among the adult population in the United States.

## Methods

### Study design and participants

We conducted a secondary analysis using data from the publicly accessible NHANES database (https://www.cdc.gov/nchs/nhanes/index.htm). The NHANES, conducted every 2 years by the National Center for Health Statistics (NCHS) in the United States, evaluates the health and nutritional status of adults and children through a combination of structured interviews, physical examinations, and laboratory tests. NHANES evaluates approximately 5000 individuals across the United States each year using a comprehensive two-stage process. First, participants complete a structured health interview that covers a wide range of topics, including their current and past medical conditions, access to healthcare, and lifestyle factors. This interview, conducted by trained field interviewers, typically takes about 1 h and is conducted in-person or via telephone. Following the interview, participants undergo a free health examination at a Mobile Examination Center (MEC). The health examination is tailored to each participant based on their age and gender and is conducted by highly trained health professionals. It includes measurements such as height, weight, blood pressure, oral health, vision, hearing, and bone density, as well as laboratory tests for environmental exposures, kidney and liver function, and allergies or sensitivities to various substances. These examinations ensure high-quality and standardized data collection. The entire process, including the interview and examination, typically requires several hours to complete. The study protocol was approved by the NCHS Research Ethics Review Board, and all participants provided written informed consent.

**Fig. 1:**
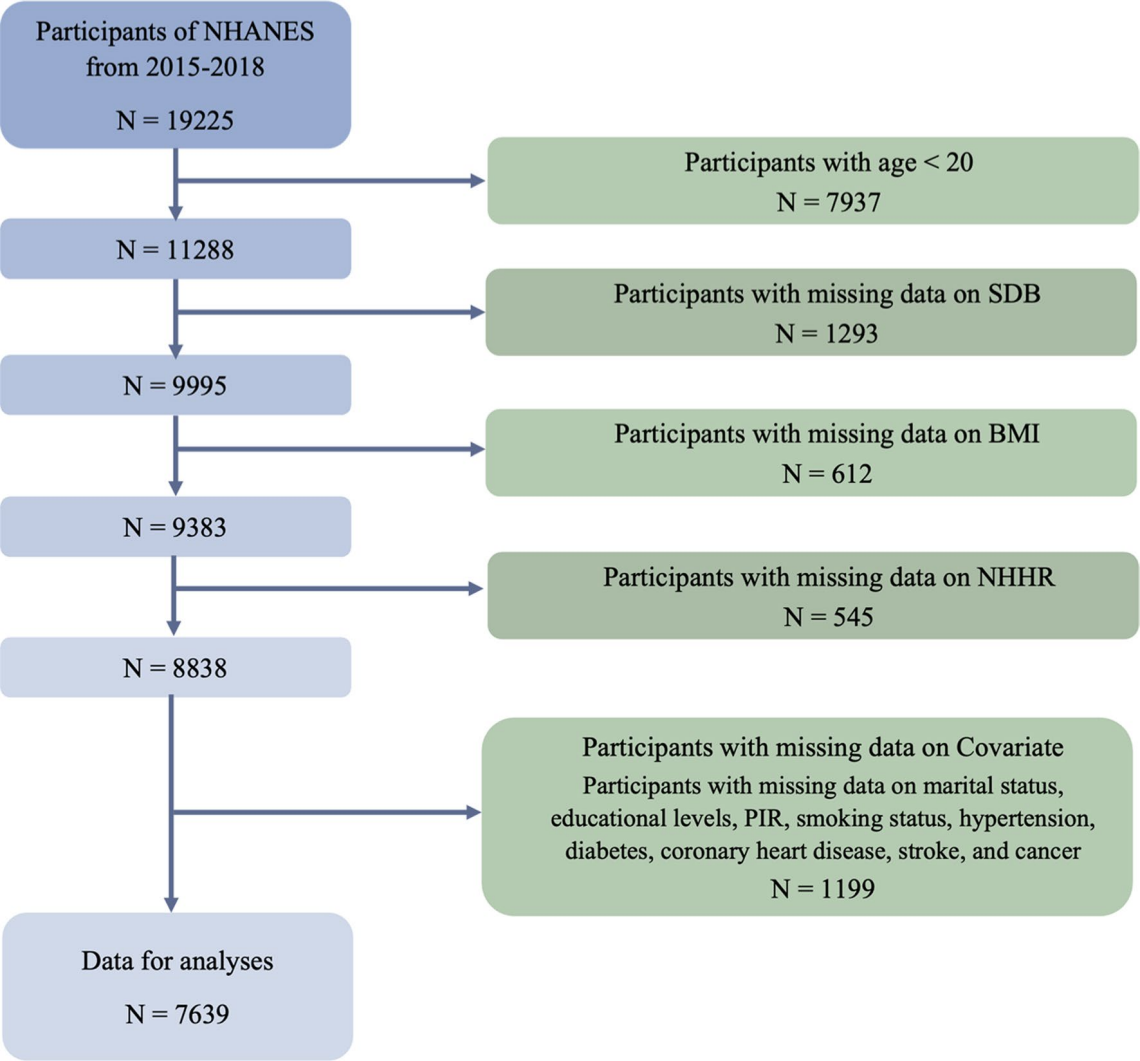
Participant selection process. *NHANES* National Health and Nutrition Examination Survey, *SDB* sleep-disordered breathing, *BMI* body mass index, *NHHR* non-high-density lipoprotein cholesterol to high-density lipoprotein cholesterol ratio, *PIR* poverty-income ratio

This study included data from the NHANES database from the 2015–2016 and 2017–2018 cycles. The participant selection process is illustrated in Fig. 1; a final sample of 7,639 participants was included in the study. The exclusion criteria were as follows: (1) age < 20 years; (2) missing data on SDB, BMI (obesity indicator), or NHHR (mediating variable); and (3) missing covariate data, including marital status, educational levels, poverty–income ratio (PIR), smoking status, hypertension, diabetes, coronary heart disease, stroke, and cancer.

Table 1 presents the unweighted characteristics of the study population. Out of 7639 participants, 3900 (51.05%) exhibited symptoms of SDB. Among those with SDB symptoms, the BMI distribution was as follows: < 25, 709 (18.18%) individuals; 25–30, 1242 (31.85%) individuals; and ≥ 30, 1949 (49.97%) individuals. The remaining 3739 participants, who did not exhibit SDB symptoms, showed a different BMI distribution: < 25, 1357 (36.31%) individuals; ≥ 25 and < 30, 1222 (32.68%) individuals; and ≥ 30, 1160 (31.01%) individuals. These values are not weighted, providing a direct representation of the sample characteristics.

**Table 1:** Unweighted characteristics of the study population

Characteristic	Total *N* = 7639	Without SDB *N* = 3739			With SDB *N* = 3900		
		BMI < 25 *N* = 1357	BMI ≥ 25, < 30 *N* = 1222	BMI ≥ 30 *N* = 1160	BMI < 25 *N* = 709	BMI ≥ 25, < 30 *N* = 1242	BMI ≥ 30 *N* = 1949
*Age*							
Mean (SD)	49.05 (17.42)	43.93 (18.33)	49.15 (18.41)	48.11 (17.48)	50.28 (17.97)	53.46 (16.05)	49.86 (15.74)
Median [Min, Max]	49.00 [20.00, 80.00]	40.00 [20.00, 80.00]	48.00 [20.00, 80.00]	48.00 [20.00, 80.00]	52.00 [20. 00, 80.00]	55.00 [20.00, 80.00]	50.00 [20.00, 80.00]
*Sex*							
Male	3711 (48.6%)	555 (40.9%)	616 (50.4%)	460 (39.7%)	378 (53.3%)	752 (60.5%)	950 (48.7%)
Female	3928 (51.4%)	802 (59.1%)	606 (49.6%)	700 (60.3%)	331 (46.7%)	490 (39.5%)	999 (51.3%)
*Race*							
Mexican American	1163 (15.2%)	105 (7.7%)	211 (17.3%)	188 (16.2%)	57 (8.0%)	219 (17.6%)	383 (19.7%)
Non-Hispanic Black	863 (11.3%)	107 (7.9%)	143 (11.7%)	124 (10.7%)	58 (8.2%)	181 (14.6%)	250 (12.8%)
Non-Hispanic White	2710 (35.5%)	490 (36.1%)	448 (36.7%)	422 (36.4%)	263 (37.1%)	414 (33.3%)	673 (34.5%)
Other Hispanic	1562 (20.4%)	252 (18.6%)	220 (18.0%)	307 (26.5%)	120 (16.9%)	195 (15.7%)	468 (24.0%)
Other race	1341 (17.6%)	403 (29.7%)	200 (16.4%)	119 (10.3%)	211 (29.8%)	233 (18.8%)	175 (9.0%)
*Marital status*							
Married	4108 (53.8%)	637 (46.9%)	645 (52.8%)	574 (49.5%)	402 (56.7%)	768 (61.8%)	1082 (55.5%)
Widowed	464 (6.1%)	75 (5.5%)	85 (7.0%)	76 (6.6%)	47 (6.6%)	70 (5.6%)	111 (5.7%)
Divorced	746 (9.8%)	112 (8.3%)	116 (9.5%)	120 (10.3%)	63 (8.9%)	113 (9.1%)	222 (11.4%)
Separated	233 (3.1%)	42 (3.1%)	33 (2.7%)	39 (3.4%)	14 (2.0%)	44 (3.5%)	61 (3.1%)
Never married	1336 (17.5%)	358 (26.4%)	214 (17.5%)	234 (20.2%)	115 (16.2%)	139 (11.2%)	276 (14.2%)
Living with partner	752 (9.8%)	133 (9.8%)	129 (10.6%)	117 (10.1%)	68 (9.6%)	108 (8.7%)	197 (10.1%)
*Education levels*							
Less than high school	1510 (19.8%)	232 (17.1%)	244 (20.0%)	210 (18.1%)	141 (19.9%)	287 (23.1%)	396 (20.3%)
High school	1724 (22.6%)	268 (19.7%)	270 (22.1%)	288 (24.8%)	158 (22.3%)	274 (22.1%)	466 (23.9%)
More than high school	4405 (57.7%)	857 (63.2%)	708 (57.9%)	662 (57.1%)	410 (57.8%)	681 (54.8%)	1087 (55.8%)
*PIR*							
Mean (SD)	2.55 (1.61)	2.65 (1.63)	2.58 (1.62)	2.45 (1.60)	2.56 (1.63)	2.61 (1.63)	2.47 (1.57)
Median [Min, Max]	2.14 [0, 5.00]]	2.34 [0, 5.00]	2.18 [0, 5.00]	2.03 [0, 5.00]	2.10 [0, 5.00]	2.21 [0, 5.00]	2.10 [0, 5.00]
*Smoking*							
Yes	3163 (41.4%)	468 (34.5%)	458 (37.5%)	450 (38.8%)	324 (45.7%)	571 (46.0%)	892 (45.8%)
No	4476 (58.6%)	889 (65.5%)	764 (62.5%)	710 (61.2%)	385 (54.3%)	671 (54.0%)	1057 (54.2%)
*Hypertension*							
Yes	2673 (35.0%)	229 (16.9%)	369 (30.2%)	479 (41.3%)	199 (28.1%)	471 (37.9%)	926 (47.5%)
No	4966 (65.0%)	1128 (83.1%)	853 (69.8%)	681 (58.7%)	510 (71.9%)	771 (62.1%)	1023 (52.5%)
*Diabetes*							
Yes	1135 (14.9%)	69 (5.1%)	152 (12.4%)	213 (18.4%)	74 (10.4%)	183 (14.7%)	444 (22.8%)
No	6504 (85.1%)	1288 (94.9%)	1070 (87.6%)	947 (81.6%)	635 (89.6%)	1059 (85.3%)	1505 (77.2%)
*Coronary heart disease*							
Yes	328 (4.3%)	29 (2.1%)	50 (4.1%)	59 (5.1%)	32 (4.5%)	60 (4.8%)	98 (5.0%)
No	7311 (95.7%)	1328 (97.9%)	1172 (95.9%)	1101 (94.9%)	677 (95.5%)	1182 (95.2%)	1851 (95.0%)
*Stroke*							
Yes	294 (3.8%)	36 (2.7%)	39 (3.2%)	53 (4.6%)	31 (4.4%)	45 (3.6%)	90 (4.6%)
No	7345 (96.2%)	1321 (97.3%)	1183 (96.8%)	1107 (95.4%)	678 (95.6%)	1197 (96.4%)	1859 (95.4%)
*Cancer*							
Yes	699 (9.2%)	106 (7.8%)	126 (10.3%)	101 (8.7%)	59 (8.3%)	134 (10.8%)	173 (8.9%)
No	6940 (90.8%)	1251 (92.2%)	1096 (89.7%)	1059 (91.3%)	650 (91.7%)	1108 (89.2%)	1776 (91.1%)
*BMI (kg/m*^*2*^*)*							
Mean (SD)	29.59 (7.16)	21.91 (2.07)	27.28 (1.42)	35.57 (5.58)	22.47 (1.87)	27.54 (1.41)	36.70 (6.18)
Median [Min, Max]	28.50 [14.20, 86.20]	22.20 [14.20, 24.90]	27.20 [25.00, 29.90]	33.80 [30.00, 68.20]	22.90 [15.50, 24.90]	27.60 [25. 00, 29.90]	34.90 [30.00, 86.20]
*Total cholesterol (mg/dL)*							
Mean (SD)	189.35 (41.48)	184.09 (38.96)	191.17 (42.83)	188.47 (43.52)	186.33 (38.94)	193.82 (41.39)	190.63 (41.59)
Median [Min, Max]	186.00 [77.00, 545.00]	180.00 [77.00, 362.00]	188.00 [85.00, 416.00]	185.00 [81.00, 540.00]	183.00 [93.00, 328.00]	191.50 [79.00, 545.00]	188.00 [84.00, 433.00]
*HDL-C (mg/dL)*							
Mean (SD)	53.79 (16.61)	62.94 (17.26)	54.11 (16.77)	50.64 (14.78)	59.92 (18.09)	52.26 (15.64)	47.84 (13.34)
Median [Min, Max]	51.00 [6.00, 226.00]	61.00 [18. 00, 149.00]	51.00 [19. 00, 226.00]	48.00 [6. 00, 151.00]	57.00 [26.00, 178.00]	49.00 [23.00, 189.00]	46.00 [16.00, 112.00]
*NHHR*							
Mean (SD)	2.82 (1.47)	2.11 (1.02)	2.80 (1.35)	3.01 (1.72)	2.34 (1.14)	3.00 (1.41)	3.27 (1.55)
Median [Min, Max]	2.56 [0.21, 27.00]	1.90 [0.36, 9.81]	2.55 [0.20, 12.83]	2.73 [0.58, 27.00]	2.13 [0.40, 8.37]	2.78 [0.36, 15.03]	2.98 [0.60, 20.833]

*SDB* sleep-disordered breathing, *NHHR* non-high-density lipoprotein cholesterol to high-density lipoprotein cholesterol ratio, *HDL-C* high-density lipoprotein cholesterol, *TC* total cholesterol, *PIR* poverty–income ratio, *BMI* body mass index, *SD* standard deviation

### Assessment of SDB

In this study, variables related to SDB were derived from the Sleep Disorders Questionnaire file (SLQ), which is part of the NHANES questionnaire data set. The acronym "SLQ" refers to the “Sleep Disorders” data file, which is publicly accessible on the NHANES website. For the 2015–2016 cycle, the file is labeled as SLQ_I, and for the 2017–2018 cycle, it is labeled as SLQ_J. The definition of SDB was based on the guidelines proposed by the US Department of Health and Human Services [18] and was supported by previous research [19]. The conditions for SDB included self-reports of the following: (1) snoring ‘3 nights or more a week’ in response to SLQ030; (2) snorting or stopping breathing ‘3 nights or more a week’ in response to SLQ040; and (3) sleeping for ‘7 h or more’ on weekdays or workdays in response to SLD012, combined with feeling ‘excessively or overly sleepy during the day’ ‘almost always to 16–30 times a month’ in response to SLQ120. Based on these criteria, the SDB symptoms were coded as follows: 0 = no and 1 = yes.

### Assessment of BMI

BMI variables were obtained from the body measurement file (BMX_Doc) of the examination data. BMI was calculated by trained professionals who measured the participants' height and weight. BMI was determined by dividing weight (kg) by the square of height (m). Based on the World Health Organization BMI classification standards [20] and previous studies [21, 22], BMI was classified as follows: (1) underweight or normal, < 25.0 kg/m^2^, (2) overweight, 25.0–29.9 kg/m^2^, and (3) obese, ≥ 30.0 kg/m^2^.

### Assessment of NHHR

The NHHR variables in this study were obtained from the cholesterol files (HDL_Doc and TCHOL_Doc) of the laboratory data. The ‘HDL_Doc’ file provided data on HDL-C levels (mg/dL), and the ‘TCHOL_Doc’ file, on total cholesterol (TC) levels (mg/dL). The NHHR was determined by subtracting HDL-C from TC and dividing the result by HDL-C [23].

### Covariates

Several covariates were considered in this study: age (continuous variable), sex (male or female), race, marital status, educational level, and poverty–income ratio (PIR). Smoking status was defined as having smoked at least 100 cigarettes in one's lifetime. The presence of hypertension, diabetes, coronary heart disease, stroke, or cancer was recorded as a binary variable (yes or no).

### Statistical analysis

Data were weighted according to the NHANES standard sample weighting guidelines to ensure the representativeness of the sample. First, we used the chi-square test with Rao–Scott second-order corrections to compare the sample characteristics of participants with and without SDB. The weighted Wilcoxon rank-sum test was used for the continuous variables.

We employed multivariate logistic regression models to examine the association between BMI and SDB and presented the results as odds ratios (OR) with 95% confidence intervals (CI). Three models were constructed to adjust for confounders. Model 1 was a crude model without covariate adjustments. Model 2 (the partially adjusted model) was adjusted for age, sex, race, educational level, marital status, and PIR. Model 3 (the fully adjusted model) was further adjusted for TC, smoking status, hypertension, diabetes, coronary heart disease, stroke, and cancer. We used the variance inflation factor (VIF) to test for collinearity among all variables and found that all VIF values were < 1.4, indicating no collinearity issues. In addition, we analysed the dose–response relationship between BMI and the risk of SDB using a restricted cubic spline (RCS) analysis and conducted subgroup analyses for different populations.

Next, we explored the association between the NHHR, BMI, and SDB. Multivariate linear regression was used to analyse the association between BMI and the NHHR, whereas multivariate logistic regression was used to investigate the link between the NHHR and SDB.

To elucidate whether the NHHR mediates the association between BMI and SDB, mediation analyses were conducted across the three models. We evaluated the indirect effects, direct effects, total effects, mediated proportions, and P values.

Finally, to evaluate the importance of the variables, we performed least absolute shrinkage and selection operator (Lasso) regression and logistic regression and applied the Boruta algorithm. All statistical analyses were performed using R (version 4.2; R Foundation for Statistical Computing, Vienna, Austria), with two-sided *P* values < 0.05 considered statistically significant.

## Results

### Participant characteristics

Table 2 presents the weighted characteristics of the study population. The average age of the participants was 47.21 ± 16.86 years, with 3,711 (48%) being men. The mean BMI was 29.51 ± 7.01 kg/m^2^. Notably, participants with SDB had a significantly higher BMI than those without SDB (31.38 ± 7.15 vs. 27.79 ± 6.42 kg/m^2^, *P* < 0.001). There were also significant differences in age, sex, race, marital status, education level, smoking, hypertension, diabetes, coronary heart disease, stroke, TC, HDL-C, and NHHR between the two groups (*P* < 0.05).

**Table 2:** Characteristics of the study population (weighted)

Characteristic	Total *N* = 179,397,459^a^	Without SDB *N* = 91,267,666^a^	With SDB *N* = 88,129,793^a^	*P* value^b^
Age	47.21 ± (16.86)	45.13 ± (17.36)	49.35 ± (16.04)	< 0.001
Sex				< 0.001
Male	3711 (48%)	1631 (43%)	2080 (54%)	
Female	3928 (52%)	2108 (57%)	1820 (46%)	
Race				0.031
Mexican American	1163 (8.6%)	504 (7.7%)	659 (9.5%)	
Non-Hispanic Black	863 (6.2%)	374 (5.7%)	489 (6.7%)	
Non-Hispanic White	2710 (65%)	1360 (66%)	1350 (65%)	
Other Hispanic	1562 (10%)	779 (10%)	783 (10%)	
Other race	1341 (9.8%)	722 (10%)	619 (9.0%)	
Marital status				< 0.001
Married	4108 (57%)	1856 (54%)	2252 (61%)	
Widowed	464 (4.5%)	236 (4.5%)	228 (4.5%)	
Divorced	746 (8.9%)	348 (8.5%)	398 (9.2%)	
Separated	233 (2.3%)	114 (2.3%)	119 (2.3%)	
Never married	1336 (17%)	806 (21%)	530 (13%)	
Living with partner	752 (9.9%)	379 (10%)	373 (9.7%)	
Education levels				0.011
Less than high school	1510 (11%)	686 (9.9%)	824 (13%)	
High school	1724 (24%)	826 (23%)	898 (25%)	
More than high school	4405 (65%)	2227 (67%)	2178 (62%)	
PIR	3.10 ± (1.63)	3.14 ± (1.64)	3.07 ± (1.62)	0.294
Smoking				< 0.001
Yes	3163 (42%)	1376 (37%)	1787 (48%)	
No	4476 (58%)	2363 (63%)	2113 (52%)	
Hypertension				< 0.001
Yes	2673 (31%)	1077 (25%)	1596 (37%)	
No	4966 (69%)	2662 (75%)	2304 (63%)	
Diabetes				< 0.001
Yes	1135 (11%)	434 (8.3%)	701 (14%)	
No	6504 (89%)	3305 (92%)	3199 (86%)	
Coronary heart disease				0.020
Yes	328 (4%)	138 (2.9%)	190 (4.4%)	
No	7311 (96%)	3601 (97%)	3710 (96%)	
Stroke				0.013
Yes	294 (3%)	128 (2.2%)	166 (3.2%)	
No	7345 (97%)	3611 (98%)	3734 (97%)	
Cancer				0.402
Yes	699 (10%)	333 (9.9%)	366 (11%)	
No	6940 (90%)	3406 (90%)	3534 (89%)	
BMI status				< 0.001
< 25	2066 (27%)	1357 (37%)	709 (18%)	
≥ 25, < 30	2464 (32%)	1222 (33%)	1242 (31%)	
≥ 30	3109 (41%)	1160 (30%)	1949 (52%)	
BMI (kg/m^2^)	29.51 ± (7.01)	27.79 ± (6.42)	31.38 ± (7.15)	< 0.001
Total cholesterol (mg/dL)	190.95 ± (41.33)	189.79 ± (41.13)	192.16 ± (41.50)	0.042
HDL-C (mg/dL)	54.47 ± (17.21)	57.38 ± (18.28)	52.01 ± (15.56)	< 0.001
NHHR	2.79 ± (1.47)	2.59 ± (1.42)	3.00 ± (1.49)	< 0.001

*SDB* sleep-disordered breathing, *NHHR* non-high-density lipoprotein cholesterol to high-density lipoprotein cholesterol ratio, *HDL-C* high-density lipoprotein cholesterol, *TC* total cholesterol, *PIR* poverty–income ratio, *BMI* body mass index

^a^*N* (unweighted) (%); Mean ± (SD)

^b^Chi-squared test with Rao & Scott's second-order correction; Wilcoxon rank-sum test for complex survey samples

### Association between BMI and SDB

Table 3 shows the association between BMI and SDB. The weighted multivariate logistic regression results indicated a positive correlation between BMI and SDB, which remained significant across all models. When BMI was treated as a continuous variable, each unit increase in BMI was associated with an 8% increase in the risk of SDB in the fully adjusted model, Model 3 (OR: 1.08, 95% CI: 1.06–1.10, *P* < 0.001).

**Table 3:** Association between BMI and SDB (weighted)

Exposure	Model 1		Model 2		Model 3	
	OR (95% CI)	*P* value	OR (95% CI)	*P* value	OR (95% CI)	*P* value
BMI	1.08 (1.06,1.10)	< 0.001	1.08 (1.06,1.10)	< 0.001	1.08 (1.06,1.10)	< 0.001
BMI status						
< 25	1 (Ref.)		1 (Ref.)		1 (Ref.)	
≥ 25, < 30	1.94 (1.55,2.43)	0.001	1.68 (1.32,2.12)	< 0.001	1.65 (1.26,2.15)	0.003
≥ 30	3.52 (2.82,4.39)	< 0.001	3.24 (2.59,4.06)	< 0.001	3.09 (2.40,3.99)	< 0.001
*P* for trend		< 0.001		< 0.001		< 0.001

Model 1: Not adjusted

Model 2: Adjusted for age, sex, race, education level, marital status, and PIR

Model 3: Additionally adjusted for smoking, hypertension, diabetes, TC, coronary heart disease, stroke, and caner.

*BMI* body mass index, *SDB* sleep-disordered breathing, *OR* odds ratio, *CI* confidence interval

Furthermore, we categorised patients into three groups based on BMI and compared the risk of SDB among these groups using multivariate logistic models. The results showed a significant positive correlation between increased BMI and the risk of SDB in all models. In the fully adjusted model (Model 3), participants with a BMI of 25.0 ≤ BMI < 30.0 kg/m^2^ and those with a BMI of ≥ 30 kg/m^2^ had a significantly higher likelihood of experiencing SDB than those with a BMI of < 25.0 kg/m^2^. Specifically, the odds were 1.65 times higher in the 25.0 ≤ BMI < 30.0 kg/m^2^ group (OR: 1.65, 95% CI: 1.26–2.15, *P* = 0.003) and 3.09 times higher in the ≥ 30 kg/m^2^ group (OR: 3.09, 95% CI: 2.40–3.99, *P* < 0.001).

### RCS analysis

**Fig. 2:**
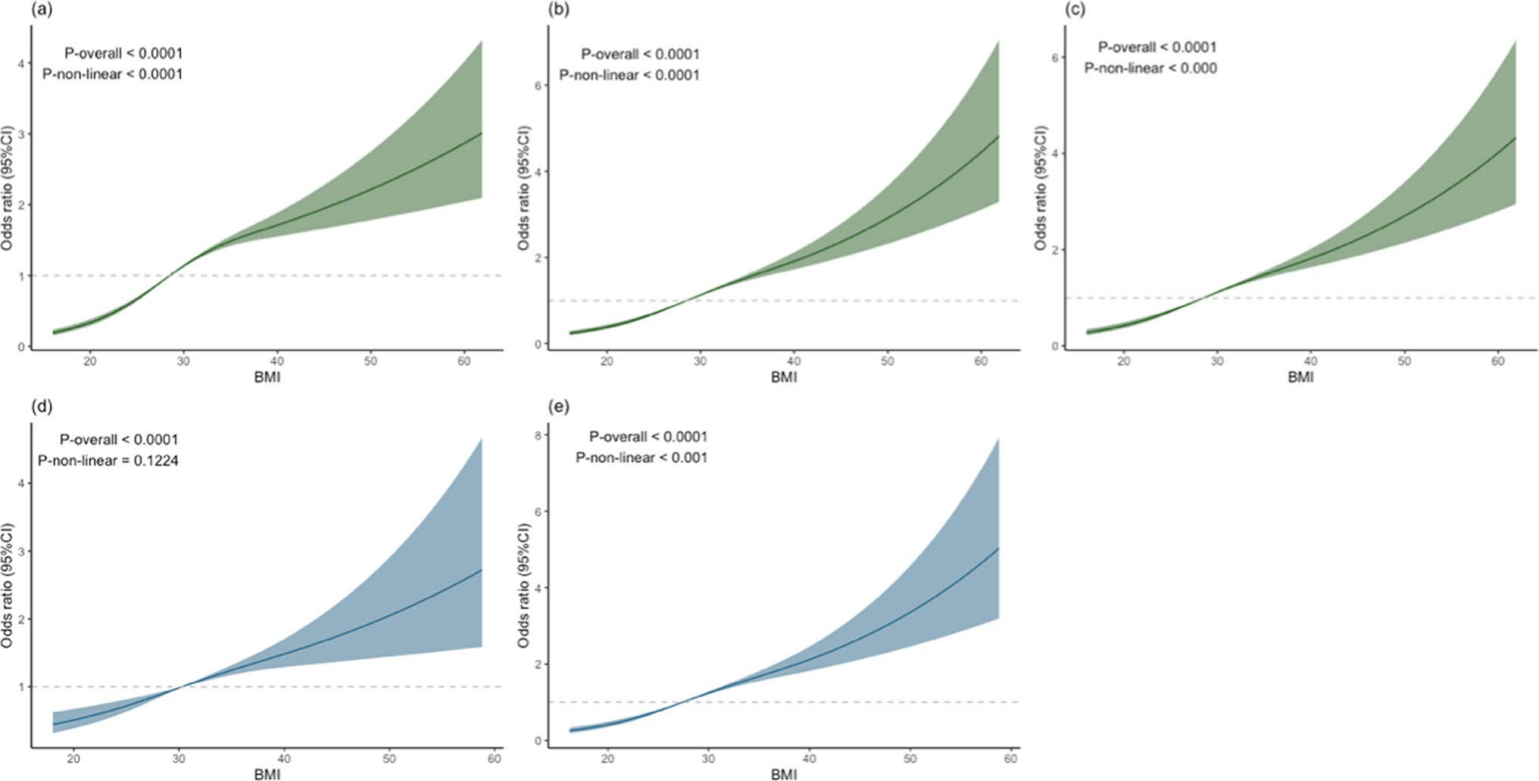
Nonlinear relationship between BMI and SDB. *NHHR* non-high-density lipoprotein cholesterol to high-density lipoprotein cholesterol ratio, *BMI* body mass index, *SDB* sleep-disordered breathing

Based on the principle of minimising the Akaike Information Criterion, three knots were selected for RCS fitting. Figure 2a–c shows the results indicating a nonlinear relationship between BMI and SDB in Models 1 (*P* for nonlinearity < 0.001), 2 (*P* for nonlinearity < 0.001), and 3 (*P* for nonlinearity < 0.001). Figure 2d illustrates that, among patients with hypertension, there was a positive linear relationship between BMI and SDB (*P* for nonlinearity = 0.1224). Figure 2e shows a nonlinear relationship between BMI and SDB in nonhypertensive patients (*P* for nonlinearity < 0.001). This suggests that BMI may have a greater impact on SDB in nonhypertensive patients than in those with hypertension.

### Subgroup analyses

**Fig. 3:**
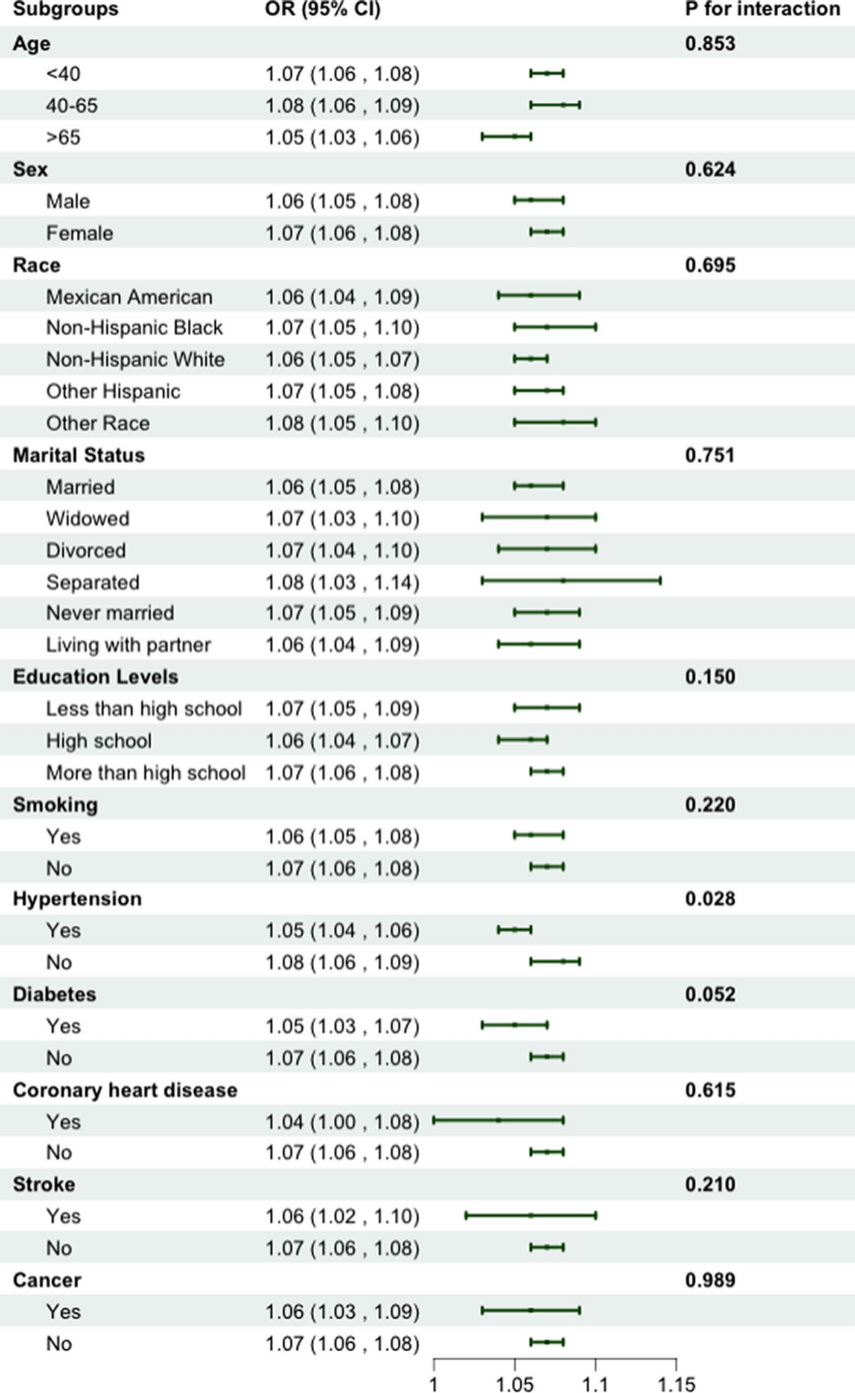
Association between BMI and SDB by different group stratifications (weighted). *BMI* body mass index, *SDB* sleep-disordered breathing, *OR* odds ratio, *CI* confidence interval

Subgroup analyses (weighted) were conducted to evaluate the association between BMI and the risk of SDB across different subgroups. As shown in Fig. 3, 11 stratification factors were included in the subgroup analyses. The results indicated no significant within-group differences in the stratification factors other than hypertension (*P* > 0.05). However, the positive correlation between BMI and SDB was stronger among nonhypertensive participants (OR: 1.08, 95% CI: 1.06–1.09, interaction *P* = 0.028). This indicates that nonhypertensive patients are more susceptible to changes in BMI, leading to SDB, which is consistent with the RCS results.

### Association of the NHHR with BMI and SDB

Table 4 shows the association between BMI and the NHHR. The results of the weighted multivariate linear regression indicated a significant positive correlation that was consistent across all models. When BMI was analysed as a continuous variable, each unit increase in BMI corresponded to a 5% rise in the NHHR in the fully adjusted model (Model 3) (β = 1.05, 95% CI: 0.04–0.05, *P* < 0.001).

**Table 4:** Association between BMI and the NHHR (weighted)

Exposure	Model 1		Model 2		Model 3	
	β (95% CI)	*P* value	β (95% CI)	*P* value	β (95% CI)	*P* value
BMI	0.05 (0.05, 0.06)	< 0.001	0.05 (0.05, 0.06)	< 0.001	0.05 (0.04, 0.05)	< 0.001
BMI status						
< 25	Ref.		Ref.		Ref.	
≥ 25, < 30	0.76 (0.65, 0.87)	< 0.001	0.66 (0.54, 0.77)	< 0.001	0.45 (0.36, 0.55)	< 0.001
≥ 30	1.10 (0.99, 1.20)	< 0.001	1.10 (0.96, 1.20)	< 0.001	0.78 (0.78, 0.96)	< 0.001
*P* for trend		< 0.001		< 0.001		< 0.001

Model 1: Not adjusted

Model 2: Adjusted for age, sex, race, education level, marital status, and PIR

Model 3: Additionally adjusted for smoking, hypertension, diabetes, TC, coronary heart disease, stroke, and caner.

*NHHR* non-high-density lipoprotein cholesterol to high-density lipoprotein cholesterol ratio, *SDB* sleep-disordered breathing, *OR* odds ratio, *CI* confidence interval

When BMI was considered as a categorical variable, the weighted multivariable linear regression results indicated a positive correlation between the NHHR and BMI categories. Compared to participants in the < 25.0 kg/m^2^ group, those in the 25.0 ≤ BMI < 30.0 kg/m^2^ and ≥ 30 kg/m^2^ groups showed a significant increase in the NHHR, which was consistent across all models. In the fully adjusted model (Model 3), the NHHR increased by 45% (β = 0.45, 95% CI: 0.36–0.55, *P* < 0.001) in the 25.0 ≤ BMI < 30.0 kg/m^2^ group and by 78% (β = 0.78, 95% CI: 0.78–0.96, *P* < 0.001) in the ≥ 30 kg/m^2^ group compared to in the < 25.0 kg/m^2^ group.

Table 5 outlines the relationship between the NHHR and SDB. Weighted multivariate logistic regression analysis revealed a positive correlation between the NHHR and risk of SDB in all models. When the NHHR was considered a continuous variable, each unit increase in the NHHR corresponded to a 23% greater likelihood of SDB in the fully adjusted model (Model 3) (OR: 1.23, 95% CI: 1.14–1.32,* P* < 0.001).

**Table 5:** Association between the NHHR and SDB (weighted)

Exposure	Model 1		Model 2		Model 3	
	OR (95% CI)	*P* value	OR (95% CI)	*P* value	OR (95% CI)	*P* value
NHHR	1.23 (1.17,1.30)	< 0.001	1.18 (1.11,1.26)	< 0.001	1.23 (1.14,1.32)	< 0.001
NHHR quartile						
Quartile 1	1 (Ref.)		1 (Ref.)		1 (Ref.)	
Quartile 2	1.42 (1.14,1.75)	0.002	1.35 (1.08,1.69)	0.012	1.38 (1.06,1.80)	0.024
Quartile 3	1.64 (1.30,2.07)	< 0.001	1.47 (1.16,1.87)	0.004	1.55 (1.18,2.03)	0.007
Quartile 4	2.52 (2.09,3.03)	< 0.001	2.20 (1.79,2.69)	< 0.001	2.44 (1.90,3.13)	< 0.001
*P* for trend		< 0.001		< 0.001		< 0.001

Quartile 1: 0.2045455–1.8247807, Quartile 2: 1.8247808–2.5625000, Quartile 3: 2.5625001–3.5000000, Quartile 4: 3.5000001–27.0000000

Model 1: Not adjusted

Model 2: Adjusted for age, sex, race, education level, marital status, and PIR

Model 3: Additionally adjusted for smoking, hypertension, diabetes, TC, coronary heart disease, stroke, and caner.

*NHHR* non-high-density lipoprotein cholesterol to high-density lipoprotein cholesterol ratio, *SDB* sleep-disordered breathing, *OR* odds ratio, *CI* confidence interval

To further understand this association, the NHHR (continuous) was divided into four discrete quartiles, and weighted multivariable logistic regression was used to compare the risk of SDB across the different quartiles. The findings indicated that participants in the second, third, and fourth quartiles had a significantly higher risk of SDB than those in the first quartile; this relationship remained consistent across all models. In the fully adjusted model (Model 3), the likelihood of SDB increased by 1.38 times for the second quartile (OR: 1.38, 95% CI: 1.06–1.80, *P* = 0.024), 1.55 times for the third quartile (OR: 1.55, 95% CI: 1.18–2.03, *P* = 0.007), and 2.44 times for the fourth quartile (OR: 2.44, 95% CI: 1.90–3.13, *P* < 0.001) compared to for the first quartile.

### Mediating role of NHHR

Further mediation analysis was performed to examine the mediating effect of the NHHR on the relationship between BMI and the risk of SDB. Table 6 shows that the NHHR significantly mediated this relationship. In Model 1, the NHHR explained 8.12% of the association (P < 0.001); in Model 2, 6.09% of the association (P < 0.001); and in Model 3, 5.44% of the association (P < 0.001).

**Table 6:** Mediating effects of the NHHR on the association between BMI and risk of SDB

NHHR	Indirect effects	Direct effects	Total effects	Mediated proportion (%)	*P* value
	β (95% CI)	β (95% CI)	β (95% CI)		
Model 1	0.0007 (0.0004, 0.0010)	0.0075 (0.0070, 0.0079)	0.0082 (0.0078, 0.0089)	8.12%	< 0.001
Model 2	0.0005 (0.0002, 0.0007)	0.0076 (0.0072, 0.0081)	0.0081 (0.0074, 0.0089)	6.09%	< 0.001
Model 3	0.0004 (0.0001, 0.0008)	0.0079 (0.0074, 0.0083)	0.0083 (0.0075, 0.0091)	5.44%	< 0.001

*NHHR* non-high-density lipoprotein cholesterol to high-density lipoprotein cholesterol ratio, *BMI* body mass index, *SDB* sleep-disordered breathing, *CI* confidence interval

### Importance of variables

**Fig. 4:**
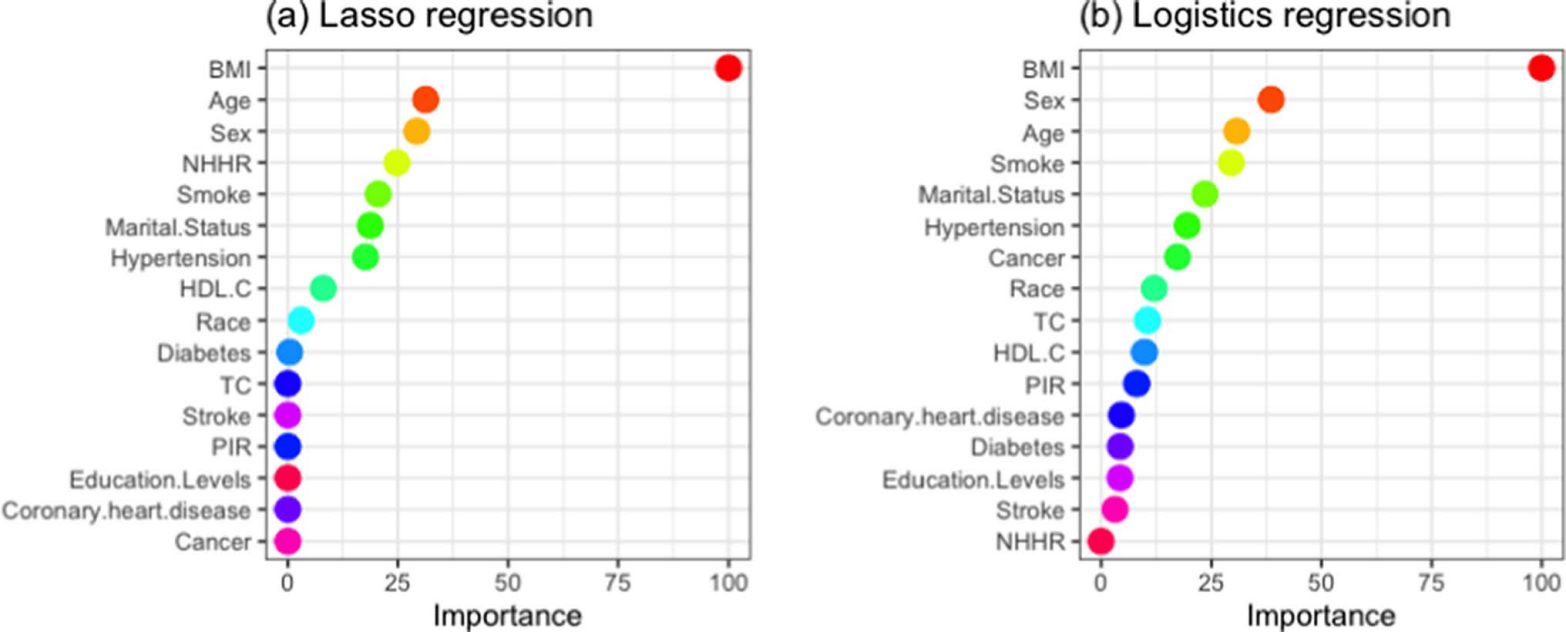
Variable importance according to **a** Lasso regression, **b** logistics regression. *BMI* body mass index, *NHHR* non-high-density lipoprotein cholesterol to high-density lipoprotein cholesterol ratio, *HDL-C* high-density lipoprotein cholesterol, *TC* total cholesterol, *PIR* poverty–income ratio

In addition, we performed Lasso and logistic regression based on the baseline data to identify the variables most closely associated with SDB. Figure 4a presents the variable importance scores from the Lasso regression, with the top three predictors being BMI, age, and sex. Figure 4b shows the variable importance scores from the logistic regression analysis, with BMI, sex, and age as the top three predictors. BMI was consistently ranked as the most important predictor of SDB in both Lasso and logistic regression.

### Boruta algorithm

**Fig. 5:**
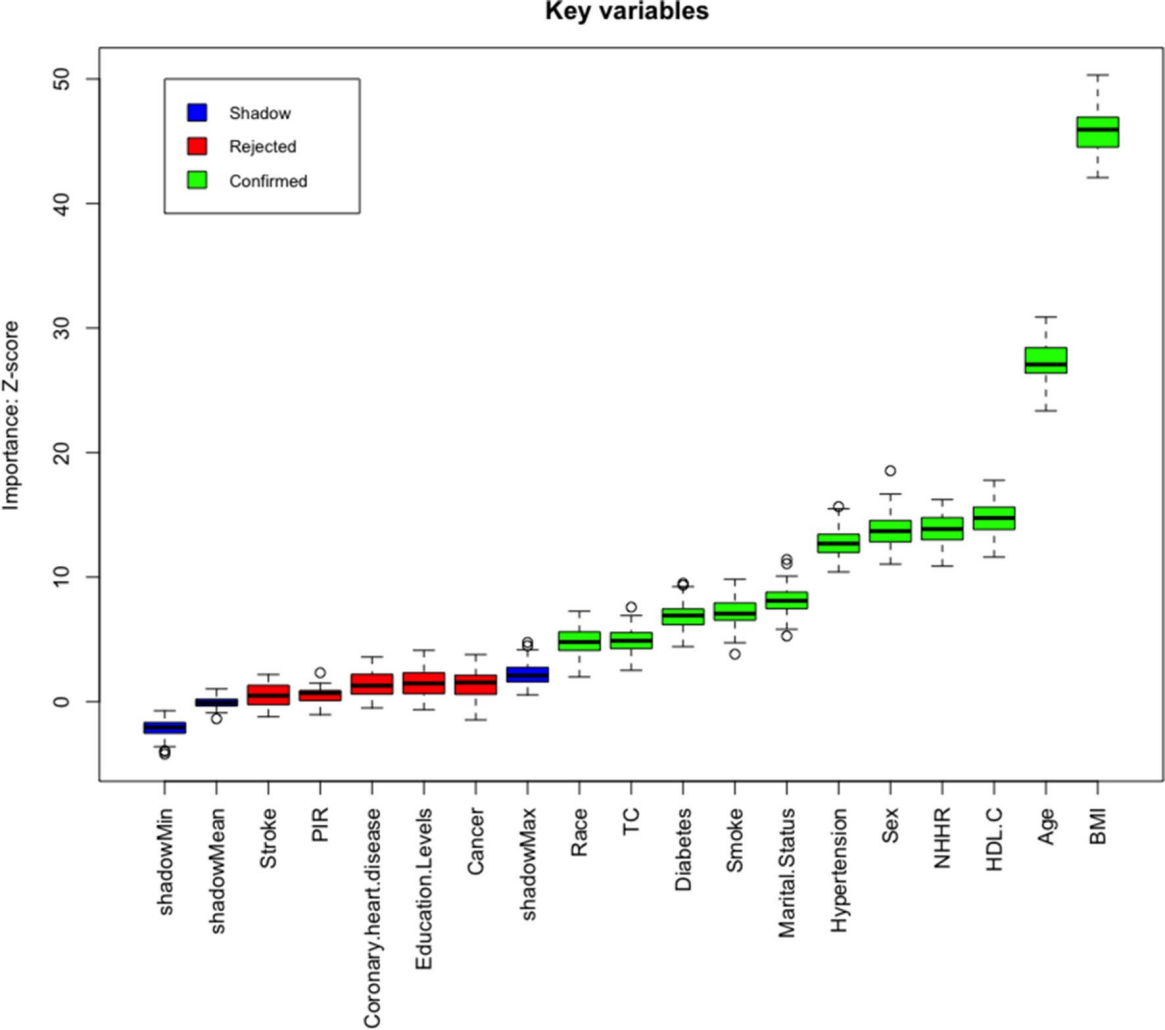
Predictor importance for SDB according to the Boruta algorithm. A predictor was deemed important if its mean importance *Z*-score was significantly higher than the maximum value of the shadow variables (blue). Conversely, a predictor (red) was excluded if its mean importance *Z*-score was significantly lower than the maximum value of the shadow variables. *SDB* sleep-disordered breathing, *NHHR* non-high-density lipoprotein cholesterol to high-density lipoprotein cholesterol ratio, *HDL-C* high-density lipoprotein cholesterol, *TC* total cholesterol, *PIR* poverty–income ratio, *BMI* body mass index

The feature selection results based on the Boruta algorithm are shown in Fig. 5. After 500 iterations, we identified the 11 variables most closely associated with SDB, ranked by their z-scores, as follows: BMI, age, HDL-C, NHHR, sex, hypertension, marital status, smoking, diabetes, TC, and race. Therefore, it can be concluded that BMI is the most important predictive factor.

## Discussion

In this comprehensive study involving 7639 American adults, a significant positive correlation was found between BMI and SDB, which was consistent across various models and analytical methods. The RCS analysis revealed a linear dose–response relationship between BMI and SDB. Subgroup analyses further revealed that nonhypertensive individuals were more susceptible to changes in BMI, leading to SDB. In addition, this study indicated that an elevated NHHR is a contributing factor to SDB, with a positive correlation between BMI and NHHR. Importantly, the mediation analysis further showed that the association between BMI and SDB was partially mediated by NHHR. Finally, the variable importance analysis confirmed the primary role of BMI in predicting SDB. In both Lasso and logistic regression, BMI was identified as the most critical predictor. These findings underscore the crucial role of obesity management in SDB prevention and treatment. The results obtained using the Boruta algorithm also supported this conclusion, ranking BMI as one of the most important predictive variables.

These findings align with those in the existing literature, further corroborating the pivotal role of obesity in SDB pathogenesis. Previous studies have indicated that obesity increases the risk of SDB by promoting upper airway fat deposition and abdominal fat accumulation, leading to airway narrowing and increased respiratory load [9]. In addition, metabolic syndrome and inflammatory responses induced by obesity are considered significant pathological mechanisms underlying the development of SDB [24, 25].

Notably, the mediation analysis revealed that the NHHR plays a significant mediating role in the association between BMI and the risk of SDB. This suggests that obesity not only has a direct effect on the development of SDB but also further increases the risk by affecting cholesterol metabolism. The NHHR, an emerging indicator, has garnered considerable attention in recent years for its role in metabolic diseases [17]. Although there is a lack of conclusive evidence on the association between the NHHR and SDB, several studies have revealed complex interactions between various cholesterol parameters and SDB. Previous research has indicated that dyslipidaemia, particularly high levels of low-density lipoprotein (LDL) and low levels of high-density lipoprotein (HDL), is closely associated with SDB severity [26, 27]. Previous studies have explored the relationship between various cholesterol parameters and SDB. For example, Bikov et al. found that low HDL-C levels significantly increased the risk of SDB [16]. Furthermore, Peracaula et al. demonstrated that dyslipidaemia exacerbates the pathological progression of SDB by promoting systemic inflammation and endothelial dysfunction [28]. These studies highlight the complex relationship between different types of cholesterol and SDB, supporting our findings on the significance of the NHHR as a mediating variable and further emphasising the role of lipid metabolism in SDB. As a comprehensive indicator, the NHHR can accurately reflect an individual's lipid metabolic state, thereby better predicting the risk of SDB.

Moreover, this study found that hypertension is an important stratifying factor, with the association between BMI and SDB being more pronounced in nonhypertensive patients. This finding is consistent with that of previous research suggesting that nonhypertensive individuals are more susceptible to developing SDB. Benjamin et al. found a complex interaction between hypertension and SDB, indicating that the vascular health of patients with hypertension may partially buffer the effect of BMI on SDB [29]. Conversely, nonhypertensive patients lacking this buffering mechanism may be more affected by increases in BMI, leading to a significantly higher risk of developing SDB. Moon et al. also suggested that the metabolic state of nonhypertensive individuals makes them more prone to apnoea and other SDB symptoms [30]. These findings underscore the importance of considering the patient’s hypertension status when assessing and managing SDB.

The clinical implications of these findings are significant. Given the strong association between BMI and SDB, healthcare providers should prioritise obesity management as a cornerstone in the prevention and treatment of SDB. Weight management strategies, including dietary modifications, physical activity, and, in some cases, bariatric surgery, could substantially reduce SDB symptoms and improve overall patient outcomes. Furthermore, the identification of NHHR as a mediating factor provides an additional tool for risk stratification. Clinicians can incorporate NHHR assessment into routine check-ups for obese patients to better predict SDB risk, enabling earlier intervention.

Future research should explore the potential for targeted therapies aimed at modifying cholesterol metabolism as an adjunct to traditional SDB treatments. For instance, investigating whether interventions that improve NHHR, such as lifestyle changes or lipid-lowering medications, could mitigate SDB severity would be valuable. In addition, longitudinal studies are needed to establish causal relationships and assess the long-term impact of NHHR on SDB progression. By bridging these gaps, future research could advance both the prevention and management of SDB, ultimately improving patient care and outcomes.

In summary, this study elucidated the significance of the NHHR as an innovative biomarker in the health domain of obesity and SDB. The NHHR effectively utilises clinically accessible and easily calculable information based on non-HDL-C and HDL-C levels, fully accounting for the impact of different cholesterol types. In addition, this study is the first to introduce the NHHR as a mediating variable and to explore its mediating role in the association between obesity and SDB. This study contributes to our understanding of the pathophysiological mechanisms between the association of obesity with SDB by providing valuable insights that could aid in early clinical diagnosis and intervention.

### Strength and limits

This study utilised representative data from a nationwide survey conducted in the United States over a 4-year period. We employed various methods to investigate the role of the NHHR in the relationship between BMI and SDB, including weighted logistic regression, RCS analysis, subgroup analyses, weighted linear regression, Lasso regression, logistic regression, and the Boruta algorithm. Our findings indicated that the NHHR mediates the relationship between BMI and SDB.

However, this study has some limitations. First, NHANES data were collected from two cycles between 2015 and 2018, potentially limiting the generalisability of the findings. Second, the cross-sectional design restricted the ability to infer causal relationships between obesity and SDB. Third, the study relied on questionnaire-based variables, which may have introduced recall bias due to self-reported data. Fourth, while BMI was used as a proxy for obesity, it is not necessarily the best estimator of body fat or obesity-related health risks. Other measures, such as fat mass or waist-to-hip ratio, may provide more precise assessments of body composition and its impact on health. Prior studies have highlighted the limitations of BMI, including its inability to differentiate between lean mass and fat mass or account for fat distribution [31, 32]. Future research could explore the role of these alternative measures in the relationship between obesity, NHHR, and SDB. Finally, despite thorough adjustments for numerous confounding factors, unknown confounders may have influenced the observed associations. Consequently, the results should be interpreted with caution and objectivity.

## Conclusions

This study confirmed that the NHHR partially mediates the association between BMI and SDB, providing new insights into the association between lipid metabolism disorders, obesity, and SDB. However, these findings require further analysis in future studies to explore potential underlying mechanisms.

### What is already known on this subject?

Obesity is a key risk factor for SDB, mainly due to increased upper airway fat deposition and respiratory load. However, the pathways linking obesity to SDB remain unclear. Limited research has examined whether obesity-induced metabolic changes, particularly in lipid metabolism, contribute to SDB, emphasizing the need to explore mediators like the NHHR.

### What this study adds?

This study identifies NHHR as a partial mediator in the association between BMI and SDB, explaining 5.44–8.12% of this relationship. The findings highlight the importance of managing both BMI and cholesterol levels to mitigate SDB risk. In addition, this study introduces NHHR as an innovative biomarker for risk stratification in obese patients, providing a new perspective on the metabolic pathways linking obesity and SDB and offering potential targets for early intervention and treatment.

## Data Availability

No datasets were generated or analysed during the current study.

## Abbreviations

SDB: Sleep-disordered breathing
BMI: Body mass index
NHHR: Non-high-density lipoprotein cholesterol to high-density lipoprotein cholesterol ratio
HDL-C: High-density lipoprotein cholesterol
NHANES: National Health and Nutrition Examination Survey
NCHS: National Center for Health Statistics
MEC: Mobile Examination Center
TC: Total cholesterol
PIR: Poverty–income ratio
OR: Odds ratios
CI: Confidence intervals
VIF: Variance inflation factor
Lasso: Least absolute shrinkage and selection operator
RCS: Restricted cubic spline
